# 

**Published:** 1882-05-20

**Authors:** 


### EDITORIALS AND MISCELLANEOUS.

MISTAKE.—Dr. R. L. Hinton, author of the article on "Trade-Marks" in our April issue, is put down in the index as a resident of Kentucky. It should have been Arkansas.

DARWIN DEAD.—Charles Robert Darwin, the celebrated Scientist and Evolutionist, died in England, on April the 20th, 1882, aged 74.

THE American Medical Association meets in St. Paul, Minn., on June 6th, 1882. President Woodward having gone to Europe, Vice-President Hooper, of Arkansas, will preside.

THE Louisiana Medical Society failed to hold its regular meeting on March the 29th, by reason of the floods. It is probable the President will call the Society together at an early day.

NO PRIZE.—The sum of $98 was subscribed to the fund for the prize on Indigenous Medicines offered by the last Georgia Medical Association, but no paper worthy of a prize being presented, the money was returned to the donors.

The Association offered a prize of $50 for the best essay on *Practice*, *Obstetrics*, *or Hygiene*.

THE Banquet given at the Markham House by the Profession of the city, to the members of the State Medical Association, was a sumptuous and brilliant affair. The tables, the decorations, the toasts, the social enjoyment and the array of medical talent were fully up to any occasion of the kind which has occurred in Atlanta.

The reception at the Governor's Mansion and at Mr. Porter's, and also the excursion up the Georgia Pacific Railroad, were interesting features of the present Association, and will long be remembered with pleasure by those fortunate members of the profession who participated in them.

EXPULSION OF DR. HEERY.—It will be noticed in our sketch of the proceedings of the late meeting of the Georgia Medical Association, that Dr. D. O. C. Heery was expelled from the Association, by reason of advertising a specialty. Whatever may be said upon the ethical question involved, that provision of the Constitution of the Association which gives the Board of Censors plenary power to expel a member without the vote or ratification of the body as a whole, is certainly arbitrary and despotic, and contrary to the fundamental and time-honored principles of our government, which guarantee a hearing and right of trial even to the most abandoned culprit. We trust that a motion to modify or repeal this law will prevail at the next meeting of the Association.

### *RECEIPTED.*

1881.—Drs. J. P. Stevens, M. L. Mahaffy, Joseph Jones.

1882.—Drs. Z. T. Young, T. A. Gibson, L. W. Coleman, M. D. Miles, W. A. Culbertson, M. V. B. Miller, J. F. Earnest, S. J. Ellis, W. T. McConnell, Jas. F. Brooks, E. L. Sloan, T. T. Smith, J. Myers, M. T. Grant, S. S. Potter, E. B. Kitcherside, to September, 1882, M. V. Am[illegible]

